# Cardiac troponin T quantitative assay failure as a result of antibody interference

**DOI:** 10.4102/ajlm.v2i1.23

**Published:** 2013-12-03

**Authors:** Philip H. Fortgens, Fierdoz Omar

**Affiliations:** 1 Department of Clinical Laboratory Sciences, Division of Chemical Pathology, University of Cape Town, South Africa; 2 National Health Laboratory Service, Groote Schuur Hospital, South Africa

## Abstract

**Background:**

Immunoassays are prone to interference by various substances which may cause inaccurate results. This type of interference is difficult to detect analytically.

**Objective:**

A case of CARDIAC Troponin T Quantitative reader (Roche Diagnostics) assay failure was detected and investigated in order to ascertain the likely cause.

**Method:**

Patient whole blood was mixed with cardiac troponin T-positive blood, patient and control sera were denuded of immunoglobulin G by protein A-affinity chromatography and patient sera were mixed with mouse serum. Samples were analysed on a CARDIAC Troponin T Quantitative reader.

**Results:**

A mixture of patient whole blood and cardiac troponin T-positive blood resulted in assay failure; removal of immunoglobulin G from patient sera reversed the cardiac troponin T assay failure; the addition of mouse serum as a heterophile antibody blocking agent had no effect.

**Conclusion:**

It is proposed that the interference resulting in assay failure may not be because of a heterophile antibody, but rather a result of a circulating autoantibody to cardiac troponin T, which may compete with antibody assay reagents for binding sites.

## Introduction

A consensus document released by the European Society of Cardiology, the American College of Cardiology, the American Heart Association and the World Heart Federation task force in 2012 has proposed cardiac troponin (cTn) as the preferred biomarker for myocardial necrosis because of its superlative myocardial tissue specificity and high clinical sensitivity.^1^ Furthermore, cTn has also been shown to have value for the prediction of adverse cardiovascular events in patients presenting with acute coronary syndrome.^2^ Cardiac troponin T (cTnT) appears to be an important marker of coronary heart disease, mortality and risk of heart failure in a healthy population without manifest cardiovascular disease.^3^

Measurement of cardiac troponins is achieved by immunoassay. Despite extensive experience with this methodology, however, immunoassays are occasionally subject to interfering substances that compromise their accuracy – indeed, it is estimated that antibody interference affects approximately one in 2000 immunoassay results.^4^ We report a novel case of assay failure using the CARDIAC Troponin T Quantitative reader (Roche Diagnostics).

## Research method and design

### Case

**FIGURE 1:**
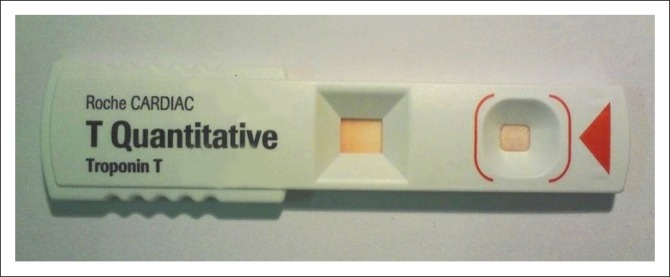
Absence of a control line on the Roche CARDIAC Troponin T Quantitative test strip.

A 61-year-old female, with a history of ischaemic heart disease and hypertension, presented to the emergency unit on two occasions 12 days apart with chest discomfort. Repeated attempts by the diagnostic laboratory to obtain cTnT measurements failed, as reflected by the absence of a positive control line on test strips (CARDIAC Troponin T Quantitative reader, Roche Diagnostics; Figure 1). As the creatinine kinase level was within normal limits (26–140 U/L) at both visits and the myoglobin was normal (7–64 ng/L) when measured at the second visit, the patient was discharged with follow-up.

### Interference experiments

Antibody interference was suspected and the following investigation was thus performed. Prior ethics approval was not obtained as the investigation would lead to improvement in this patient’s management. Firstly, a 1:1 mixture of the patient’s sample and a recently-assayed anonymous sample positive for cTnT (both heparinised whole bloods), was assayed for cTnT.^5^ Secondly, patient and control plasma samples were depleted of immunoglobulin G (IgG) using protein A-affinity chromatography.^6^ These samples were analysed for cTnT prior to and after IgG depletion. The CARDIAC Troponin T Quantitative reader is a lateral flow immunoassay, utilising the sandwich principle on a test strip with two murine monoclonal anti-cTnT antibodies.^7^ Thirdly, in order to exclude the presence of interfering human anti-mouse antibodies (HAMA), mouse serum was added to the patient plasma (1:4) and the mixture was incubated for one hour at room temperature, following which the cTnT was measured. Lastly, to determine whether the automated cTnT assay on the Roche Elecsys E170 analyser was subject to the same interference, dilutions of a known cTnT-positive plasma sample mixed with the patient plasma were assayed for cTnT.

## Results

The mixture of whole blood patient sample and a cTnT-positive specimen inhibited the formation of the control line on the cTnT reagent strip, supporting our suspicion of an interfering substance. Whilst only the control sample elicited a control line prior to IgG depletion (cTnT < 0.03 ng/ml), both the patient and control samples elicited control lines after IgG depletion (cTnT < 0.03 ng/ml), suggesting that IgG was the interfering substance. Test-strips contain HAMA-blocking antibodies,^7^ but despite the presence of additional blocking agent (mouse serum), the control line did not develop, which suggested strongly that the interfering IgG was not an HAMA (Table 1). Dilutions of a known cTnT-positive plasma sample with the patient plasma showed a linear response when assayed for cTnT on the Roche Elecsys E170 analyser, suggesting that this platform is not subject to the same autoantibody interference.

**TABLE 1:** CARDIAC Troponin T Quantitative test strip performance.

Sample tested	Control line
Patient whole blood	Absent
cTnT-positive whole blood	Present
Patient whole blood + cTnT-positive whole blood (1:1)	Absent
Protein A-affinity chromatography: control serum	Present
Protein A-affinity chromatography: patient serum	Present
Mouse blocking serum + patient plasma	Absent

cTnT, cardiac troponin T.

## Discussion

There is increasing use of cTn measurement in the diagnosis of myocardial injury and any inaccuracy with regard to measured values is more likely to have serious clinical impact than would be the case with immunoassays for many other analytes. Several immunochemical interferences have been well described, including heterophile antibodies,^8^ rheumatoid factor^9^ and circulating troponin autoantibodies.^10,11^ Recent studies have shown that 15.9% of samples from cohorts of normal blood donors were positive for autoantibodies to troponin T or troponin I and 10.9% were positive for both.^12^ Autoantibody–antigen macrocomplexes have been well described for a number of biomarkers such as salivary amylase,^13^ creatine kinase,^14^ aspartate aminotransferase^15^ and lactate dehyrogenase,^16^ but there is no evidence to suggest that these complexes contribute to a pathological process. In contrast, cTn autoantibodies have been shown to contribute to the progression toward heart failure in mice and a similar process may occur in humans.^17^ Furthermore, it has been proposed that the reduced clearance of immunoglobulin– cardiac troponin complexes from the circulation may result in increased levels of measured troponins.^18^ This phenomenon does not, therefore, represent assay interference, but rather an analytically-true result, albeit misleading.

**FIGURE 2:**
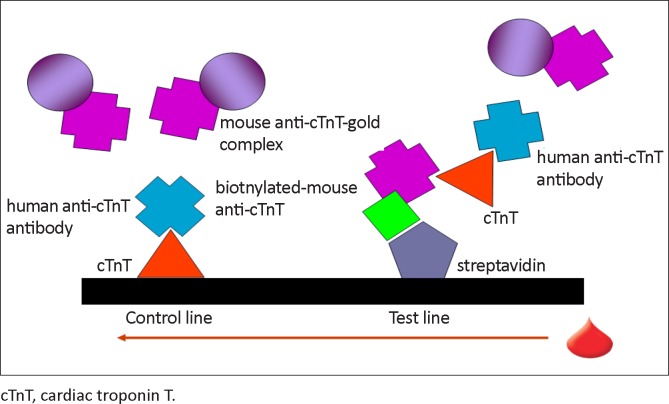
Possible effect of blocking human anti-cardiac troponin T antibodies on the Roche CARDIAC Troponin T Quantitative lateral flow immunoassay.

This study shows that the removal of IgG from patient serum reverses the cTnT assay failure and that the addition of mouse serum as a heterophile antibody-blocking agent has no effect. This, together with the quantifiable result of the myoglobin assay (Roche Diagnostics), which uses the same test principle, species of monoclonal antibody and reader as the cTnT assay, suggests that the interference may not be because of a heterophile antibody, but rather because of an autoantibody to cTnT. Such an antibody may compete with the mouse anti-cTnT–gold complex for binding to immobilised cTnT on the test-strip, which normally serves as a test control. In the case of the myoglobin assay, the immobilised control antigen (myoglobin) is different, which may explain why it is unaffected. An autoantibody could also compete with reagent anti-cTnT antibodies for binding sites, thereby preventing the development of a test line (Figure 2).

There have been several reports of positive interference in the cTnT assay by heterophilic antibodies with the CARDIAC Troponin T Quantitative test^7^ and the Elecsys E170^8^ automated platform but, to the best of our knowledge, this is the first report of cTnT assay failure on the former platform. We conclude that the failure of this assay is a result of putative autoantibodies to cTnT. Assay failure also allowed for the immediate detection of potential interference, but the majority of cases of interference remain undetected at the analytical level. It is crucial, therefore, that any discrepancies between results and the clinical picture be addressed by clinicians with the laboratory, to avoid further potentially-invasive and expensive investigations.

## Conclusion

A case of assay failure was detected in the CARDIAC Troponin T Quantitative reader. Immunoassays are subject to interference and it is important to investigate anomalous results to exclude such assay interference. After removing IgG from patient serum and re-assaying the cTnT, assay failure was found to reverse. This case highlights the fact that immunoassay interference remains a persistent problem and vigilance is encouraged in order to minimise its impact. Furthermore, in the case of troponins, interference may be caused by circulating autoantibodies to cTn.
